# 新辅助靶向治疗在*EGFR*突变可手术切除肺腺癌患者中的应用价值

**DOI:** 10.3779/j.issn.1009-3419.2025.106.18

**Published:** 2025-07-20

**Authors:** Shijie HUANG, Mengying FAN, Kaiming PENG, Wanpu YAN, Boyang CHEN, Wu WANG, Tianbao YANG, Keneng CHEN, Mingqiang KANG, Jinbiao XIE

**Affiliations:** ^1^351100 莆田，莆田学院附属医院胸心外科一区; ^1^Department I of Cardiothoracic Surgery, The Affiliated Hospital of Putian University, Putian 351100, China; ^2^100142 北京，北京市癌症与转化研究重点实验室，北京大学肿瘤医院胸外一科; ^2^Key Laboratory of Carcinogenesis and Translational Research (Ministry of Education/Beijing), Department I of Thoracic Surgery, Peking University Cancer Hospital & Institute, Beijing 100142, China; ^3^350000 福州，福建医科大学附属协和医院胸外二科; ^3^Department II of Thoracic Surgery, Fujian Medical University Union Hospital, Fuzhou 350000, China; ^4^100142 北京，国家分子肿瘤学重点实验室，北京市癌症与转化医学研究重点实验室，北京大学肿瘤医院胸外一科; ^4^State Key Laboratory of Molecular Oncology, Beijing Key Laboratory of Carcinogenesis and Translational Research, Department I of Thoracic Surgery, Peking University Cancer Hospital & Institute, Beijing 100142, China

**Keywords:** 肺肿瘤, 新辅助靶向治疗, 表皮生长因子受体, 主要病理缓解, Lung neoplasms, Neoadjuvant targeted therapy, Epidermal growth factor receptor, Major pathologic response

## Abstract

**背景与目的** 我国非小细胞肺癌（non-small cell lung cancer, NSCLC）患者中表皮生长因子受体（epidermal growth factor receptor, *EGFR*）突变占比较高，然而这部分患者当前的新辅助治疗方案缺乏明显获益。本研究旨在通过分析新辅助靶向治疗在*EGFR*突变可手术切除肺腺癌患者中的有效性和安全性，探索其潜在的应用价值。**方法** 采用多中心回顾性研究的方法，分析2019年7月至2024年10月接受新辅助靶向治疗后进行手术切除的*EGFR*突变IIA-IIIB期肺腺癌患者的治疗效果。**结果** 共纳入来自3个中心的24例*EGFR*突变肺腺癌患者，所有患者均成功接受手术，且均实现R0切除（100.0%）。客观缓解率（objective response rate, ORR）为83.3%（20/24），主要病理缓解率（major pathologic response, MPR）为37.5%（9/24），其中2例（8.3%）患者实现了病理完全缓解（pathological complete response, pCR）。在新辅助治疗期间，24例患者中有13例（54.2%）经历了1-2级不良事件，未出现3级及以上不良事件。皮疹（4例，16.7%）、口腔溃疡（2例，8.3%）、腹泻（2例，8.3%）是最常见的治疗相关不良事件。中位随访时间为33.0个月，所有患者均未出现死亡，总生存率为100.0%，1、2年无病生存率分别为91.1%和86.2%。**结论** 新辅助单药靶向治疗在*EGFR*突变可手术切除肺腺癌患者中的应用是安全且可行的，有望成为*EGFR*突变肺腺癌患者的一种极具前景的新辅助治疗方案。

## Abstract (en)

**Background and objective** The proportion of patients with non-small cell lung cancer (NSCLC) harboring epidermal growth factor receptor (*EGFR*) mutations is relatively high in China. However, these patients currently lack significant benefits from available neoadjuvant treatment options. This study aims to explore the potential application value of neoadjuvant targeted therapy by evaluating its efficacy and safety in patients with *EGFR*-mutant resectable lung adenocarcinoma. **Methods** A multicenter retrospective study was used to analyze the treatment effect of patients with stage IIA-IIIB *EGFR*-mutant lung adenocarcinoma who underwent surgical resection after receiving neoadjuvant targeted therapy from July 2019 to October 2024. **Results** A total of 24 patients with *EGFR*-mutant lung adenocarcinoma from three centers were included in this study. All patients successfully underwent surgery and achieved R0 resection of 100.0%. The objective response rate (ORR) was 83.3% (20/24). The major pathologic response (MPR) rate was 37.5% (9/24), with 2 patients (8.3%) achieving pathological complete response (pCR). During neoadjuvant therapy, 13 out of 24 patients (54.2%) experienced adverse events of grade 1-2, with no occurrences of ≥ grade 3. The most common treatment-related adverse events were rash (*n*=4, 16.7%), mouth sores (*n*=2, 8.3%), and diarrhea (*n*=2, 8.3%). The median follow-up time was 33.0 months, no deaths occurred in all patients, and the overall survival (OS) rate was 100.0%. The 1-year disease-free survival (DFS) rate was 91.1%, and the 2-year DFS rate remained at 86.2%. **Conclusion** The application of neoadjuvant targeted therapy in patients with *EGFR*-mutant resectable lung adenocarcinoma is safe and feasible, and is expected to become a highly promising neoadjuvant treatment option for the patients with *EGFR*-mutant lung adenocarcinoma.

肺癌是全球所有癌症中死亡率及发病率最高的恶性肿瘤^[1]^。非小细胞肺癌（non-small cell lung cancer, NSCLC）在所有肺癌中约占85%，其中肺腺癌是最常见的组织病理学类型，约占NSCLC的55%^[2]^。临床上约1/3 NSCLC患者被诊断时已处于局部进展期（III期）^[3]^。对于局部进展期NSCLC，首选治疗方式是手术切除，但是预后并不理想，II-IIIA期患者的5年总生存（overall survival, OS）率为36%-60%，术后局部复发和远处转移是导致患者死亡的主要原因^[4]^。因此，国内外肺癌诊疗指南均推荐采用以术前新辅助治疗联合手术切除为主的多学科综合治疗模式作为可切除局部晚期NSCLC的主要治疗手段^[5,6]^。

相较于辅助治疗缺乏可测量病灶和有效的疗效检测方式，新辅助治疗可直接通过观察病灶大小变化来判断药物敏感性，同时具备缩小病灶并消除隐匿性微转移，从而提高根治性切除的可能性，以及改善患者预后等优势^[7]^。目前临床上，可切除NSCLC患者仍主要采用新辅助化疗的方式，然而并未取得令人满意的疗效，仅在手术基础上提高了5%的5年OS率^[8]^。随着免疫治疗的进一步发展，新辅助化疗联合免疫治疗显著改善了驱动基因阴性NSCLC的预后，但对驱动基因阳性的NSCLC效果并不理想。

我国NSCLC患者中的驱动基因阳性占比较高，尤其以表皮生长因子受体（epidermal growth factor receptor, *EGFR*）突变为著，40.0%-57.7%的NSCLC包含*EGFR*基因突变^[9]^。*EGFR*突变与患者对靶向治疗的敏感性密切相关，已有研究^[10]^表明不同突变类型在治疗反应上存在显著差异。随着靶向治疗在*EGFR*阳性NSCLC的治疗过程中的效果得到证实，无论是在局部晚期NSCLC的术后辅助治疗中，还是在晚期NSCLC患者群体中，均展现出显著的生存获益^[11,12]^。因此，越来越多的临床医师开始关注酪氨酸激酶抑制剂（tyrosine kinase inhibitors, TKIs）治疗在*EGFR*突变NSCLC患者的新辅助治疗中的应用前景。然而，目前对新辅助靶向治疗在*EGFR*突变肺腺癌中应用的相关研究仍较少。

本研究旨在通过多中心回顾性研究分析2019年7月至2024年10月接受新辅助靶向治疗的*EGFR*突变IIA-IIIB期肺腺癌患者的临床特征、EGFR-TKIs治疗方案、治疗疗效、治疗相关不良事件以及预后生存等资料，评估新辅助靶向治疗在局部晚期肺腺癌患者中的疗效、安全性以及可能的生存优势，为该治疗策略在临床实践中的应用提供依据。

## 1 资料与方法

### 1.1 临床资料

回顾性分析2019年7月至2024年10月就诊于北京大学肿瘤医院、莆田学院附属医院、福建医科大学附属协和医院的24例接受新辅助靶向治疗后进行手术切除的*EGFR*突变IIA-IIIB期肺腺癌患者。患者在接受手术切除前，依据驱动基因类型给予相应的靶向药物治疗，包括第一、二、三代EGFR-TKIs治疗。其中，北京大学肿瘤医院16例，莆田学院附属医院6例，福建医科大学附属协和医院2例。本研究伦理声明遵循《赫尔辛基宣言》（2013年修订版）。本文为回顾性研究，经莆田学院附属医院伦理审查委员会审查批准并免除患者知情同意（伦理批件号：莆医附伦[2025165]），所有参与机构均已知悉并同意此项研究。

### 1.2 纳入标准与排除标准

纳入标准：（1）年龄>18岁；（2）经组织病理学确诊为肺腺癌；（3）*EGFR*基因检测显示18-G719X、19-Del、20-ins、21-L858R基因突变；（4）治疗前经胸部增强计算机断层扫描（computed tomography, CT）或正电子发射型计算机断层显像（positron emission tomography/CT, PET/CT）、头颅磁共振成像（magnetic resonance imaging, MRI）、全身骨扫描、腹部彩超/腹部CT检查除外脑、骨、肝、肾上腺等转移，并证实临床分期为IIA-IIIB期（T2b-4N0M0、T1-4N1-2M0）[根据第8版国际抗癌联盟（Union for International Cancer Control, UICC）NSCLC肿瘤原发灶-淋巴结-转移（tumor-node-metastasis, TNM）分期标准]；（5）美国东部肿瘤协作组（Eastern Cooperative Oncology Group, ECOG）评分0-1分；（6）既往无恶性肿瘤病史。排除标准：（1）无明确组织学或细胞学诊断；（2）既往接受过化疗或其他相关抗肿瘤治疗；（3）有严重心脏、肝脏或肾脏等疾病，不能耐受手术或预期生存时间不长者；（4）临床资料不完整。

### 1.3 主要观察指标

收集各种临床数据，如患者临床基础资料、影像学和病理学缓解反应评估、手术相关指标、药物不良事件和预后结果。

疗效评估方面关注患者新辅助治疗后影像学缓解率。依据实体瘤疗效评价标准（Response Evaluation Criteria in Solid Tumors, RECIST）分为完全缓解（complete response, CR）、部分缓解（partial response, PR）、疾病稳定（stable disease, SD）、疾病进展（progressive disease, PD）。客观缓解率（objective response rate, ORR）包括CR和PR。病理学缓解率可分为病理完全缓解（pathological complete response, pCR）、主要病理缓解（major pathological response, MPR）、部分病理缓解，MPR是指术后标本病理检测残留肿瘤细胞≤10%。预后评价包括无病生存期（disease-free survival, DFS）和OS等。DFS定义为从手术日到客观证据证实疾病进展或任何原因引起复发、进展或死亡的时间。OS定义为从新辅助靶向治疗第1天到任何原因导致死亡时间。若到研究截止时间疾病未进展或未死亡的患者，以最后一次肿瘤随访评估的日期进行计算。

手术相关指标包括手术切除程度、手术方式、手术时间、术中失血量以及术后住院时间等详细信息。药物相关毒性反应依据不良事件常用术语标准（Common Terminology Criteria for Adverse Events, CTCAE）进行分级。

### 1.4 随访

通过门诊复诊或电话交谈进行随访。靶向治疗期间依据患者影像学缓解程度每1-3个月复查评效；手术后第1年内每3个月复查胸部CT、肿瘤标志物，随后2-5年内每半年复查胸部CT平扫+增强，每年复查1次颅脑MRI、腹部超声、全身骨显像扫描等检查，或者根据医生的判断必要时进行。最后一次随访时间为2025年3月31日。

### 1.5 统计学方法

采用统计软件SPSS 26.0处理数据。计数资料以计数和频数（%）表示，两组间比较总体样本量≤24例，故组间比较采用费舍尔精确检验（*Fisher’s* exact test）。计量资料以均数±标准差（Mean±SD）或中位数和四分位距（interquartile range, IQR）表示。采用*Kaplan-Meier*法绘制DFS曲线。*P*<0.05为差异有统计学意义。

## 2 结果

### 2.1 患者基本特征

2019年7月至2024年10月共有24例符合标准的患者被纳入研究，病理类型均为肺腺癌。所有患者均接受了新辅助靶向治疗后进行手术切除的治疗模式，并且术后均继续口服原方案靶向药物治疗，有2例术后病理为pCR的患者未继续靶向治疗。24例患者中，中位年龄为60.0岁（IQR：57.0-67.8岁），13例患者（54.2%）为女性，58.3%的患者未曾吸烟，22例患者（91.7%）的ECOG评分为0分。本研究中部分患者合并有基础疾病，包括高血压病7例，2型糖尿病2例，心律失常2例，脑梗死病史2例，慢性阻塞性肺疾病1例。

根据全身相关检查结果，24例患者进行了术前临床分期，具体分布为：1例IIA期（4.2%）、12例IIB期（50.0%）、10例IIIA期（41.6%）、1例IIIB期（4.2%）。在*EGFR*驱动基因突变方面，12例（50.0%）为21-L858R突变，10例（41.6%）为19-Del突变，20-ins和18-G719X突变各1例（各占4.2%）。对于19-Del和21-L858R基因突变的患者，EGFR-TKIs药物选择如下：吉非替尼6例，埃克替尼2例，奥希替尼7例，阿美替尼5例，伏美替尼2例。针对18-G719X和20-ins基因突变的2例患者，分别口服达克替尼和阿法替尼进行靶向治疗（表1）。

**表1:** 患者基线临床特征

Clinical demographics		Data (n=24)
Age (median, IQR, yr)		60.0 (57.0-67.8)
Gender	Male	11 (45.8%)
	Female	13 (54.2%)
Smoking	Former	10 (41.7%)
	Never	14 (58.3%)
ECOG performance status	0	22 (91.7%)
	1	2 (8.3%)
BMI (median, IQR, kg/m^2^)		23.7 (22.6-25.2)
Histology	Adenocarcinoma	24 (100.0%)
EGFR mutation	18-G719X	1 (4.2%)
	19-Del	10 (41.6%)
	20-ins	1 (4.2%)
	21-L858R	12 (50.0%)
Preoperative staging	PET/CT	21 (87.5%)
	EBUS	3 (12.5%)
T stage	T1	6 (25.0%)
	T2	11 (45.8%)
	T3	4 (16.7%)
	T4	3 (12.5%)
N stage	N0	6 (25.0%)
	N1	10 (41.7%)
	N2	8 (33.3%)
TNM stage	IIA	1 (4.2%)
	IIB	12 (50.0%)
	IIIA	10 (41.6%)
	IIIB	1 (4.2%)

IQR: interquartile range; ECOG: Eastern Cooperative Oncology Group; BMI: body mass index; EGFR: epidermal growth factor receptor; PET/CT: positron emission tomography/computed tomography; EBUS: endobronchial ultrasound; TNM: tumor-node-metastasis.

### 2.2 疗效评价

所有肺腺癌患者均达到R0切除（100.0%）。17例患者接受了胸腔镜下肺叶切除术（75.0%），以及1例患者因肿瘤与肺门粘连紧密，在手术过程中由胸腔镜转为开胸手术。新辅助靶向治疗的中位持续给药时间为12.0周（IQR：8.0-20.0周），从新辅助治疗结束到手术的中位时间为4.0 d（IQR: 2.0-6.8 d），其中9例MPR患者的中位靶向用药时间为16周，其中2例pCR患者的用药时间分别为14与24周。中位手术时间为119.5 min（IQR: 80.3-150.0 min），中位术中出血量为50.0 mL（IQR: 50.0-100.0 mL），中位术后住院时间为4.0 d（IQR: 4.0-6.0 d），在围手术期内，术后并发症包括肺部感染2例（8.3%）、肺漏气1例（4.2%），未出现支气管吻合口瘘及任何死亡病例（表2）。

**表2:** 患者手术相关指标

Clinical demographics	Data (n=24)
Duration of neoadjuvant therapy (median, IQR, wk)	12.0 (8.0-20.0)
Time from neoadjuvant therapy completion to resection (median, IQR, d)	4.0 (2.0-6.8)
Postoperative length of stay (median, IQR, d)	4.0 (4.0-6.0)
Surgery type	
VATS wedge resection	2 (8.3%)
VATS segmentectomy	4 (16.7%)
VATS lobectomy	17 (70.8%)
Open lobectomy	1 (4.2%)
Surgical outcomes	
R0 resection	24 (100.0%)
Postoperative pulmonary infection	2 (8.3%)
Bronchial anastomotic fistula	0 (0.0%)
Pulmonary leakage	1 (4.2%)
Intraoperative blood loss (median, IQR, mL)	50.0 (50.0-100.0)
Operative duration (median, IQR, min)	119.5 (80.3-150.0)

VATS: video-assisted thoracoscopic surgery.

在进行影像学疗效评估时，尽管没有患者达到CR，但是整体ORR达到了83.3%（表3）。进一步的分层分析显示，不同的*EGFR*突变类型之间的ORR无统计学差异（*P*=0.594）。然而，*EGFR* 19-Del突变组的ORR高于*EGFR *21-L858R组（90.0% *vs* 75.0%），且*EGFR* 19-Del突变组患者治疗后达到ORR的几率是*EGFR* 21-L858R组的3倍[优势比（odds ratio, OR）=3.0，95%CI：0.26-34.58]。在不同的EGFR-TKIs药物选择组别（第一代或第三代TKIs）中，ORR亦未显示统计学差异（*P*=0.602）。然而，在第三代EGFR-TKIs治疗组中，*EGFR* 19-Del突变患者的ORR显著优于*EGFR* 21-L858R突变患者（100.0% *vs* 75.0%）。此外，第一代EGFR-TKIs治疗组达到ORR的几率仅是第三代组的0.50倍（OR=0.50, 95%CI: 0.06-4.47）（表4，图1A）。

**表3:** 新辅助靶向治疗患者疗效评价

Clinical demographics	Data (n=24)
EGFR-TKIs drug	
First-generation TKIs	8 (33.3%)
Second-generation TKIs	2 (8.3%)
Third-generation TKIs	14 (58.4%)
Radiological response	
PR	20 (83.3%)
SD	4 (16.7%)
Pathological response	
pCR (no viable tumor)	2 (8.3%)
MPR (≤10% viable tumor)	7 (29.2%)
11%-50% residual viable tumor	5 (20.8%)
>50% residual viable tumor	10 (41.7%)
Tumor downstaging	15 (62.5%)
cT4 to ≤ypT3	3 (100.0%)
cT3 to ≤ypT2	4 (100.0%)
cT2 to ≤ypT1	6 (54.6%)
cT1c to ≤ypT1a	2 (50.0%)
Lymph node downstaging	8 (44.4%)
cN2 to ypN0	3 (37.5%)
cN1 to ypN0	5 (50.0%)
Pathological downstaging	13 (54.2%)
cIIIB to ≤ypIB	1 (100.0%)
cIIIA to ≤ypIB	5 (50.0%)
cIIB to ≤ypIB	7 (58.3%)

TKIs: tyrosine kinase inhibitors; PR: partial response; SD: stable disease; pCR: pathological complete response; MPR: major pathological response.

**表4:** 不同类型EGFR突变以及接受不同EGFR-TKIs治疗患者的新辅助靶向治疗疗效

Items	EGFR					EGFR-TKIs			
	19-Del (n=10)	21-L858R (n=12)	χ^2^	P		First-generation (n=8)	Third-generation (n=14)	χ^2^	P
Radiological response									
PR	9 (90.0%)	9 (75.0%)	0.825	0.594		6 (75.0%)	12 (85.7%)	0.392	0.602
SD	1 (10.0%)	3 (25.0%)				2 (25.0%)	2 (14.3%)		
Pathological response									
pCR/MPR	6 (60.0%)	3 (25.0%)	2.764	0.192		2 (25.0%)	7 (50.0%)	1.316	0.380
Non-MPR	4 (40.0%)	9 (75.0%)				6 (75.0%)	7 (50.0%)		

**图1:**
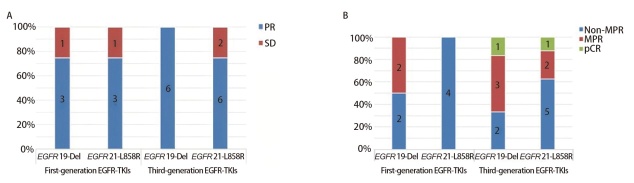
不同代际EGFR-TKIs对不同EGFR突变类型的疗效评价。A：影像学疗效评价；B：病理学疗效评价。

瀑布图（图2）显示了每例患者在接受新辅助靶向治疗后的病理学缓解情况。在病理学缓解方面，24例cT1c-4期患者中有15例（62.5%）患者的肿瘤病灶实现了病理降期；18例cN期患者中有8例（44.4%）患者实现了淋巴结降期。同时，13例（54.2%）患者达到了TNM分期的病理降期（表3）。此外，在所有患者完成手术切除后共有9例达到了MPR，MPR率为37.5%（95%CI: 18.8%-59.4%），其中pCR率为8.3%（95%CI: 1.0%-27.0%）。

**图2:**
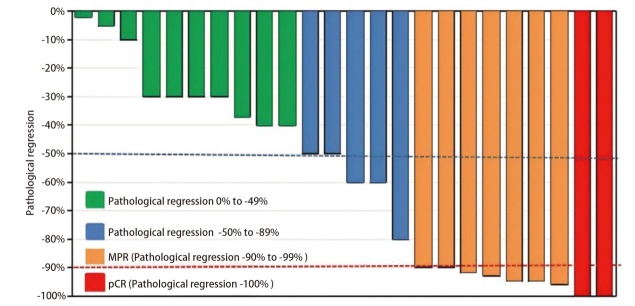
患者新辅助靶向治疗后病理学缓解率的瀑布图。红线：肿瘤病理缓解达90%；蓝线：肿瘤病理缓解达50%。

对不同*EGFR*突变类型患者的疗效差异进行深入分析，显示在*EGFR* 19-Del组患者中，有6例达到MPR，而在*EGFR* 21-L858R组患者中则有3例（60.0% *vs* 25.0%），两组之间无显著差异（*P*=0.192）。然而，*EGFR *19-Del突变组患者发生MPR的几率是*EGFR *21-L858R组的4.50倍（OR=4.50, 95%CI: 0.73-27.74）。在选择不同EGFR-TKIs药物治疗的组别中，两组之间亦无统计学差异（*P*=0.380）。在第一代EGFR-TKIs组的8例患者中，仅有2例达到MPR，且无pCR患者，而在第三代EGFR-TKIs组的14例患者中，有7例达到MPR，并有2例达到pCR（25.0% *vs* 50.0%），且第一代EGFR-TKIs组患者发生MPR的几率仅为第三代组的0.33倍（OR=0.33, 95%CI: 0.05-2.26）。综合分析表明，第三代EGFR-TKIs组患者的MPR率显著优于第一代组（表4，图1B）。

进一步的差异分析显示，尽管在影像学缓解率和病理学缓解率两个方面，不同亚组之间均未显示出显著差异，但无论在第一代还是第三代EGFR-TKIs治疗组中，19-Del突变组患者的MPR率均高于21-L858R突变组（分别为50.0% *vs* 0.0%、66.7% *vs* 37.5%）（图1B）。

### 2.3 治疗相关不良事件

根据CTCAE 5.0标准，在24例患者中共有13例（54.2%）在进行新辅助靶向治疗期间经历了不良事件。然而，所有患者未发生3级及以上不良事件，亦未出现因不良事件导致的药物剂量调整或治疗中断。相关的不良事件（表5）主要局限于1和2级，具体包括1-2级皮疹（*n*=4, 16.7%）、2级口腔溃疡（*n*=2, 8.3%）、2级腹泻（*n*=2, 8.3%）、2级血小板减少（*n*=2, 8.3%）、2级恶心（*n*=1, 4.2%）、1级食欲下降（*n*=1, 4.2%）以及1级谷草转氨酶升高（*n*=1, 4.2%）。

**表5:** 患者新辅助靶向治疗期间的相关不良事件

Items	Any grade	Grade 1	Grade 2
Rash	4 (16.7%)	1 (4.2%)	3 (12.5%)
Mouth sores	2 (8.3%)	0 (0.0%)	2 (8.3%)
Diarrhea	2 (8.3%)	0 (0.0%)	2 (8.3%)
Thrombocytopenia	2 (8.3%)	0 (0.0%)	2 (8.3%)
Nausea	1 (4.2%)	0 (0.0%)	1 (4.2%)
Decreased appetite	1 (4.2%)	1 (4.2%)	0 (0.0%)
AST elevation	1 (4.2%)	1 (4.2%)	0 (0.0%)
Total	13 (54.2%)	3 (12.5%)	10 (41.7%)

AST: aspartate transaminase.

### 2.4 预后疗效评价

24例患者在术后均按时进行了规范的随访复查，中位随访时间为33.0个月，范围为2.0-51.5个月。随访至截止时间，所有患者均未出现死亡事件，整体OS率为100.0%。1年DFS率为91.1%，2年DFS率为86.2%（图3A）。其中部分患者的随访时间相对较短，因此本研究目前尚未达到中位OS和中位DFS。

**图3:**
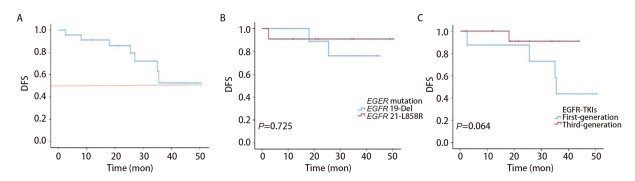
新辅助靶向治疗患者的DFS。A：整体DFS；B：不同EGFR突变类型的DFS对比；C：第一代和第三代EGFR-TKIs药物的DFS对比。

进一步的分层分析结果显示，在不同*EGFR*基因突变类型、接受不同的EGFR-TKIs药物治疗的患者中，DFS均未见显著统计学差异。然而，在接受第三代EGFR-TKIs治疗的患者组中，术后复发转移率较低，生存曲线呈现出分离趋势（*P*=0.064）（图3B、3C）。

## 3 讨论

目前根治性手术仍是NSCLC的最佳治疗手段。然而，对于局部进展期NSCLC的治疗，国内外指南一致推荐采取以手术为主的综合治疗策略，包括放化疗、靶向治疗或免疫治疗等治疗手段，以期通过缩小肿瘤体积来提高根治性切除术的可能性，以及消除潜在的微转移病灶，进而达到更好的治疗效果^[6,13]^。尽管如此，对于携带*EGFR*突变的NSCLC患者而言，最有效的术前新辅助治疗方案仍未形成共识，究竟应当优先选择靶向治疗还是化疗仍存争议^[14]^。本研究旨在探讨术前新辅助靶向治疗在*EGFR*突变可手术切除的局部进展期NSCLC中的可行性和安全性。

从外科医生的视角来看，评估新辅助治疗的疗效可以从ORR与R0切除率这两个指标直接反映出来。此外，先前关于新辅助化疗和免疫治疗的研究已经证实，MPR与患者长期生存率改善之间存在相关性^[15]^，因此，MPR率也成为了临床试验中疗效评估的一个重要替代终点。

本研究共纳入24例接受了新辅助靶向治疗的*EGFR*突变肺腺癌患者。在影像学评估方面，新辅助靶向治疗后显示出较高的ORR，整体ORR为83.3%。针对不同的EGFR-TKIs药物选择进一步分析，尽管第一代与第三代EGFR-TKIs治疗组的ORR在统计学上没有显著差异，但第三代EGFR-TKIs治疗组患者的ORR为85.7%，明显高于第一代组的75.0%。特别是在第三代EGFR-TKIs治疗组中，19-Del突变患者的ORR优于21-L858R突变患者（100.0% *vs* 75.0%）。与既往多项试验的研究结果一致，第三代EGFR-TKIs的总体ORR高于第一代或第二代，包括：EMERGING-CTONG 1103研究中厄洛替尼的54.1%^[16]^、单臂II期试验NCT01833572研究中吉非替尼的54.5%^[17]^，以及NEOS研究中奥希替尼的71.1%^[15]^、LungMate 007研究中阿美替尼的70.6%^[18]^。此外，本研究中所有患者均达到R0切除（100.0%），其数值均高于上述EGFR-TKIs新辅助靶向治疗的研究（50%-94.7%）^[16,17,19,20]^，以及化疗或放化疗的相关研究的50%-80%^[21-23]^。尽管受限于样本量，但本研究仍然显示出新辅助靶向治疗在*EGFR*突变肺腺癌中的应用优势，尤其第三代EGFR-TKIs新辅助治疗的缩瘤效果更为突出。

对于携带驱动基因阳性的患者，新辅助治疗的标准方案为化疗。尽管新辅助化疗在临床应用已久，其pCR率却大致稳定在10%的水平^[24-26]^，难以突破治疗瓶颈。本研究进一步探索了*EGFR*突变肺腺癌患者接受新辅助靶向治疗后病理缓解反应，发现新辅助靶向治疗显示出积极的结果，54.2%的患者ypTNM病理分期下降，44.4%的pN1-2期患者淋巴结受累减少。此外，9例患者（37.5%）实现了MPR，其中有2例患者（8.3%）获得pCR。这一研究结果与关于新辅助靶向治疗的研究结果大致相仿。诸如，NeoADAURA研究^[27]^中的奥希替尼单药靶向治疗组的25% MPR率（95%CI: 17%-34%）和9% pCR率（95%CI: 4%-15%）；在NEOS研究^[15]^中奥希替尼的MPR率为10.7%；在NORA研究^[28]^中奥希替尼的MPR率则为24%，以及在单臂II期试验LungMate 007研究^[18]^中阿美替尼的MPR率为21.7%，pCR率为13.0%。

值得注意的是，在上述奥希替尼的新辅助靶向治疗研究中，MPR率存在一定差异。深入分析显示，在NEOS研究^[15]^中奥希替尼的持续用药时间为6周，其MPR率仅为10.7%。然而，在NORA研究^[28]^和NeoADUARA研究^[27]^中，奥希替尼单药靶向治疗的持续时间分别延长至8-9周，这两项研究的MPR率均显著提升。结合既往关于EGFR-TKIs作为*EGFR*突变局部晚期或转移性NSCLC患者一线治疗的研究，无论第一代还是第三代EGFR-TKIs药物，其中位PFS均在9.9-22.1个月之间波动^[29-31]^，本课题组推测上述研究的新辅助靶向治疗周期可能尚未达到肿瘤病灶的最佳缓解时间。此外，本研究的新辅助靶向治疗的中位持续给药时间为12周，其中9例MPR患者的中位持续用药时间为16周，MPR率高达37.5%。这一研究结果提示，适当延长新辅助靶向治疗的持续用药时间，在一定程度上能够提高MPR率。

针对不同的EGFR-TKIs药物选择进行深入探索，尽管第一代与第三代EGFR-TKIs治疗组之间未显示出显著差异。但值得注意的是，大多数MPR患者（7/9, 77.8%）均接受了第三代EGFR-TKIs治疗，其中包括仅有的2例pCR患者。在对不同*EGFR*突变类型群体的分析中，发现携带*EGFR *19-Del突变的患者MPR率明显更高（60.0% *vs* 25.0%）。这一现象提示，携带*EGFR* 19-Del突变的局部进展期肺腺癌患者可能是新辅助靶向治疗的潜在获益人群，且接受第三代EGFR-TKIs新辅助治疗的患者疗效更佳。

从不良反应的角度评估，针对*EGFR*突变NSCLC患者，相较于含化疗的治疗方案，单药新辅助靶向治疗展现出良好的安全耐受性^[16]^。在新辅助靶向治疗期间出现的不良事件大多数为低级别，严重不良事件或者导致药物剂量调整及治疗中断的发生率较低，尤其是血液学毒性不良反应^[27,32]^。本研究中的新靶向治疗中位持续时间为12周，相较于NeoADUARA研究^[27]^中新辅助靶向治疗的周期，尽管本研究中的新辅助靶向治疗中位持续时间更长，但并未增加药物不良事件的发生率。大多数不良事件仅为轻度，未出现3级以上的不良反应，这与既往相关研究^[18,33]^结果一致。此外，本研究中中位手术时间为119.5 min，中位术中出血量为50.0 mL，中位术后住院时间为4.0 d，围手术期均未出现死亡病例。研究结果表明，术前新辅助靶向治疗不会延长手术时间、增加手术难度，也不会增加术后并发症的发生率。因此，对于*EGFR*突变肺腺癌患者，术前新辅助靶向治疗是安全且可行的，特别是对于合并基础病不耐受化疗或者不愿接受化疗的患者人群，其临床应用潜力值得进一步深入探索。

本研究结果显示，无论接受何种靶向药物治疗，所有患者均未出现死亡，OS率达到100.0%。在DFS方面，1年DFS率为91.1%，2年DFS率为86.2%。但受限于样本量及随访时间，目前尚未达到中位OS和中位DFS。通过进一步的分层分析，尽管在不同*EGFR*突变类型、EGFR-TKIs药物选择的患者之间，两组的DFS均无显著的统计学差异，但相较于第一代EGFR-TKIs治疗组，在接受第三代治疗的患者中，术后复发转移率更低，生存曲线趋势显示出长期生存的潜在优势。在EMERGING-CTONG 1103试验^[16]^中，将第一代EGFR-TKIs的新辅助治疗与新辅助化疗进行了比较。与基于铂类的双药化疗组相比，新辅助EGFR-TKIs治疗组的中位无进展生存期显著延长，为21.5个月，此外，多中心回顾性研究^[33]^结果显示，奥希替尼作为可切除*EGFR*突变NSCLC的新辅助治疗的2年OS率为89.2%，2年DFS率为87.9%。本研究受限于随访时间较短、样本量少，不足以全面评估OS，但初步数据表明新辅助靶向治疗可能对提高患者的长期生存率具有积极影响。

本研究具有一定的局限性。首先，作为一项多中心回顾性研究，样本量偏少且为单臂研究，这可能引起选择性偏倚；其次，尽管治疗前评估采用了PET/CT检查，但大多数患者未进行纵隔镜活检或EBUS引导下经支气管针吸活检进行N2分期，这可能影响N2降期率评估的准确性；最后，研究中新辅助靶向药物的选择不同、治疗持续时间的差异以及术后辅助治疗的不一致，可能导致生存数据出现偏差。后续仍需要更大规模、前瞻性的随机对照试验，进一步验证新辅助靶向治疗在提高患者长期生存率方面的确切效果。此外，未来的研究还应关注如何优化靶向药物的治疗持续时间、应用人群选择以及术后辅助治疗策略，以实现更佳的治疗效果。同时，对新辅助治疗后的肿瘤组织进行更深入的分子机制研究，将为制定更加精准和个体化的治疗方案提供重要依据。

本研究证实了新辅助单药靶向治疗在*EGFR*突变可手术切除的肺腺癌患者中的应用是切实可行且安全的，尤其是对于合并基础病不耐受化疗或者不愿接受化疗的患者人群，更具有临床应用价值。
